# Monoclonal Antibodies in Light of Mpox Outbreak: Current Research, Therapeutic Targets, and Animal Models

**DOI:** 10.3390/antib14010020

**Published:** 2025-02-26

**Authors:** Vladimir N. Nikitin, Iuliia A. Merkuleva, Dmitriy N. Shcherbakov

**Affiliations:** State Research Center of Virology and Biotechnology Vector, Rospotrebnadzor, Koltsovo 630559, Russia; nikitin_vn@vector.nsc.ru (V.N.N.); dnshcherbakov@gmail.com (D.N.S.)

**Keywords:** monoclonal antibodies, orthopoxviruses, smallpox, monkeypox, animal models

## Abstract

The rapid rise in monkeypox virus infections among humans from 2022 to 2024 has captured the attention of the global healthcare community. In light of the lack of mandatory vaccination and limited data on next-generation vaccines for monkeypox prevention, the urgent development of therapeutic agents has become a priority. One promising approach involves the use of neutralizing monoclonal antibodies. This review highlights significant advancements in the search for antibodies against human pathogenic orthopoxviruses, particularly focusing on their potential application against the monkeypox virus. We also analyze viral proteins that serve as targets for identifying therapeutic antibodies capable of neutralizing a wide range of viruses. Finally, we deemed it essential to address the challenges associated with selecting an animal model that can adequately reflect the infectious process of each orthopoxvirus species in humans.

## 1. Introduction

Orthopoxviruses (OPV) is a genus of double-stranded DNA viruses belonging to the Poxviridae family, which replicate in the cytoplasm of host cells. These viruses can infect vertebrates through various transmission routes, including airborne droplets, contact with mucous membranes, skin, or contaminated materials, as well as through scratches from infected animals [1,2]. In humans, the incubation period typically lasts 7 to 14 days. Following this period, patients commonly experience a fever and develop the onset of characteristic rashes. However, complications such as secondary bacterial infections, bronchopneumonia, sepsis, dehydration, and encephalitis can arise during the course of the illness [3].

Zoonotic orthopoxviruses pathogenic to humans have been identified in specific geographic and ecological regions across South America, Africa, Eastern Europe, the Middle East, and Asia [4,5,6,7,8]. Recent research indicates a concerning trend of annual increases in cases and an expansion of their geographical distribution [4,9]. Among these viruses, the monkeypox virus (MPXV) has emerged as a particular focus of concern due to its clinical manifestations that resemble those of Variola virus (VARV) infection and its high mortality rate. Currently, four clades of MPXV have been identified (Ia, Ib, IIa, and IIb), with clade I being the most pathogenic and associated with fatality rates of up to 10%. Since the first documented case in the Democratic Republic of the Congo in 1970 [10], MPXV cases have steadily increased and expanded beyond Africa’s borders [11].

**Figure 1:**
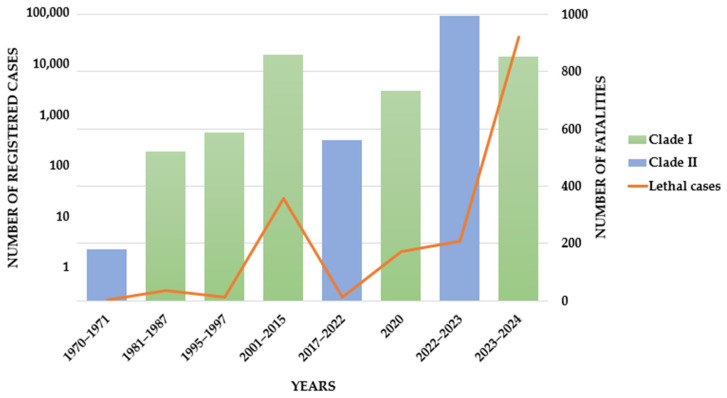
Global aggregated data on MPOX cases by clade and year. The curve shows the aggregated number of lethal cases (deaths) by years according to the date of case reporting.

The global spread of monkeypox began in 2022, and by early 2024, clade MPXV IIb had disseminated to 117 countries. By that time, over 97,000 cases had been reported worldwide, including 186 fatalities [12]. In parallel, infections from clade MPXV Ib in Africa reached unprecedented levels not seen for years, spreading throughout Central Africa by the third quarter of 2024 and even appearing in one case in Europe [13]. This alarming trend [14,15,16,17,18] (Figure 1) raises concerns about MPXV potentially invading new ecological areas and adapting to new host species [6,8].

The threat of outbreaks from other pathogenic orthopoxviruses also remains real. For instance, unaccounted live samples of VARV [19,20,21,22], as well as vaccinia virus (VACV), camelpox virus, cowpox virus (CPXV), and borealpox virus pose risks to both human health and livestock [23,24,25,26,27,28,29,30,31,32,33,34,35,36,37,38,39,40].

In response to these potential threats, a new generation of vaccines has been developed for orthopoxvirus prevention, particularly MVA-BN, which is safe for all population groups and has received emergency approval from the FDA for protecting individuals at high risk of lethal MPXV infections [41,42]. However, vaccination remains mandatory only for a very limited group of individuals whose activities involve a high risk of exposure. Therefore, multiple strategies are necessary for emergency prevention or therapy.

Over the past few decades, the administration of monoclonal antibodies (mAbs) has proven effective as both preventive and therapeutic agents. An important indication for their utilization in treating OPV infections is providing protection after the manifestation of disease symptoms. This model closely reflects the scenario of infection development and treatment in humans, where more severe symptoms of the disease course appear, placing higher demands on the tested antibodies. The high safety profile of modern mAbs makes them a viable alternative to the only immunoglobulin drug currently available for OPV infections, namely Vaccinia immune globulin (VIG).

This study aims to investigate the current research and future perspectives regarding mAbs targeting OPVs, with a particular emphasis on MPXV. By identifying specific targets within the proteomes of these viruses that can be effectively neutralized by mAbs, we seek to enhance our understanding of their potential applications in mitigating MPXV outbreaks and improving clinical outcomes. Additionally, we will explore the utilization of animal models to assess the protective properties of these antibodies, which is critical for evaluating their efficacy and safety.

As human-pathogenic OPVs continue to pose a significant public health threat—especially in light of recent MPXV outbreaks—it is essential to deepen our understanding of mAb-based therapeutic and prophylactic strategies. This knowledge will help address critical gaps in current public health measures and bolster preparedness for future viral threats.

## 2. General Characteristics of Orthopoxviruses

Orthopoxviruses are large, complex viruses ranging from 150 to 300 nm in size, characterized by their distinctive brick-shaped or oval morphology. The central core of these virions contains linear double-stranded DNA, while lateral bodies within the virion house additional proteins essential for replication; these proteins are released into the host cell cytosol during the early stages of infection.

**Figure 2:**
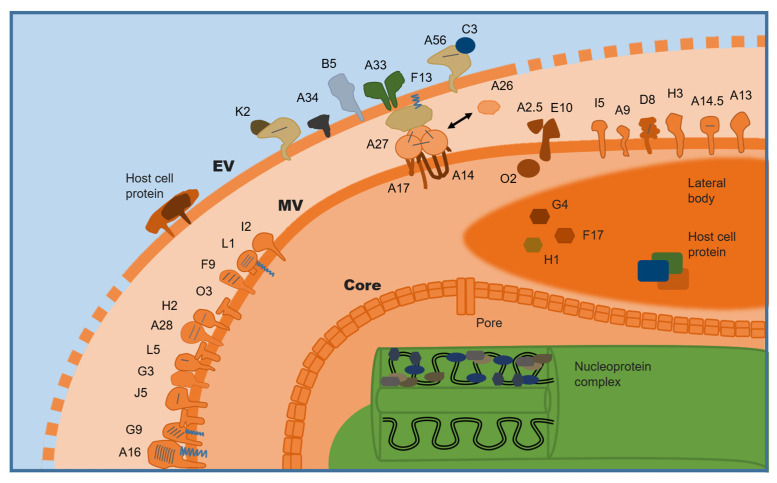
Schematic representation of structural proteins of mature (MV) and enveloped (EV) virions of orthopoxviruses. Short lines indicate disulfide bonds, while blue wavy lines represent covalently linked fatty acid molecules. The EV membrane is enriched with glycoproteins B5, A33, A34, and A56, along with the F13 protein, which plays a critical role in membrane fusion during infection and EV assembly. Protein A56 forms a complex with C3 or K2 proteins, which inhibit superinfection, thereby regulating viral propagation. Additionally, EV virions incorporate host cell-derived proteins, including cluster differentiation (CD) proteins and major histocompatibility complex (MHC) proteins, which may contribute to immune evasion or host–virus interactions.

A viral particle may possess one or two bilipid membranes, which differ in their antigenic protein composition. Orthopoxviruses exhibit two primary forms of virions: the mature virion (MV) and the enveloped virion (EV) (Figure 2).

The MV has a stable structure featuring a single bilipid envelope derived from the host cell membrane, encapsulating the viral DNA and essential proteins required for infection. This form is primarily responsible for localized, cell-to-cell spread during the initial phases of infection.

In contrast, the EV possesses an additional outer envelope acquired during the budding process, making it larger and more complex. This outer layer is enriched with glycoproteins that facilitate systemic spread, enhance immune evasion, and promote survival outside host cells, thereby enabling efficient transmission between hosts.

The OPV genome consists of a linear double-stranded DNA molecule of 170–250 kb. Covalently linked dsDNA strands form hairpin structures characterized by their AT-rich composition. Depending on the species, OPVs genomes contain 150–300 open reading frames. The central part of the genome is conservative across all OPVs; deletions and single-nucleotide substitutions are rarely observed in this region [24,43], being typically restricted to so-called hot spots [44]. Proteins encoded within this part exhibit a high degree of homology among members of the genus and family. Their functions include implementing genetic information processing, packaging of viral particles, and post-translational modification of proteins [43].

Sequences located in variable regions encode proteins that modulate cellular defense mechanisms and influence innate and specific immunity—thereby determining each virus species’ virulence and entry pathway [45,46]. These genes undergo frequent changes during viral replication through single-nucleotide substitutions and recombination [47]. Consequently, high variability among virus populations contributes to adaptation via these mechanisms. The viral genome includes genes with expression levels that remain constant throughout the replicative cycle as well as early, intermediate, and late genes transcribed at different stages [48]. Early proteins can be detected after 3 h; intermediate and late proteins can be detected after 6 h—timing varies based on multiplicity of infection and cell type. All proteins required for early gene transcription are packaged within viral particles and activated immediately after entering the cytoplasm from the nucleus. Transcription of intermediate and late genes necessitates de novo synthesis of RNA, DNA, and proteins [49].

The entry pathway of OPVs depends on pH, virus strain variations, and cell type [50], occurring either directly through the plasma membrane or via receptor-mediated endocytosis [51,52]. The infection process consists of several stages: attachment of virion to cell surface; excitation of cell membrane; fusion of viral and cellular membranes, followed by entry into the cytoplasm. The MV binding to the cell surface is mediated by four viral proteins: A26 binds laminin; A27 and H3 bind heparan sulfate; D8 binds chondroitin (Figure 2). Although these proteins are not crucial for infection, their elimination or inactivation significantly slows attachment rates [53]. Membrane fusion stages leading to core release into cytoplasm are mediated by eleven proteins forming an entry fusion complex (EFC): A16, A21, A28, F9, G3, G9, H2, J5, L1, L5, and O3 [54].

The EV entry mechanism involves an additional step requiring surface membrane exposure, potentially necessitating other cellular components for successful infection. Outer envelope disruption may occur post-endocytosis under low pH conditions or through direct interactions between viral proteins A34 and B5 with cellular glycosaminoglycans. In all cases, the disruption of the outer membrane is attributed to conformational changes in the F13 protein [55].

In contrast, the MV membrane exclusively contains viral non-glycosylated proteins. These include major transmembrane proteins A14 and A17, components of the fusion-entry complex (A28:H2, A16:G9, G3:L5), protein subcomplexes J5:F9 and H2:G9, and additional structural proteins such as L1, I5, A9, A13, and A14.5. Proteins A26, A27, H3, and D8 function as attachment factors facilitating host cell binding, while O2, A2.5, and E10 are integral to the membrane redox system required for maintaining viral integrity. Lateral bodies (LBs), located between the core and the outer membrane of MV virions, contain both viral proteins and host-derived effector molecules that are released into the host cytosol during the early stages of infection. Key LB proteins include functional glutaredoxin G4, phosphoprotein F17, and phosphatase H1, which collectively form a cytosolic redox system essential for viral replication and modulation of host cell responses.

The core of both MV and EV virions houses viral DNA along with DNA-dependent RNA polymerase and early transcription factors necessary for initiating viral gene expression immediately upon entry into the host cell. This highly organized structure ensures efficient delivery of genetic material and regulatory components to facilitate infection and replication in the host environment.

Formation of viral particles occurs through three structurally distinct stages: crescent (C), immature virion (IV), and MV [56].

The various stages of MV morphogenesis, including membranogenesis, viral DNA and protein stacking, occur exclusively within specialized membrane compartments known as virus factories. These structures are located near the endoplasmic reticulum and Golgi apparatus and are thought to form an interconnected network with these organelles [57,58,59]. The stabilization of the MV membrane is facilitated by three viral proteins: A14, A17, and D13 [60]. Additionally, a transmembrane redox system, essential for the posttranslational modification of membrane proteins in mature viral particles, is composed of three proteins: A2.5, E10, and G4 [61].

The MV membrane contains approximately 25 viral proteins but lacks cellular proteins. Instead, it is rich in phospholipids and cholesterol. A subset of MV population (1–30%) undergoes an additional envelopment stage involving a “fragile” glycoprotein membrane derived from trans-Golgi network or endosomes, leading to intracellular EV [62]. The EV membrane incorporates five specifical viral proteins: A33, A34, A56, B5, and F13. Meanwhile, the K2 and C3 proteins, which lack transmembrane domains, are associated with A56 [63]. After entering host cells, K2 and A56 remain on the surface to prevent superinfection. The A56-C3 complex further enhances complement defense mechanisms. The EV envelope contains glycoproteins crucial for systemic spread, immune evasion, and survival outside host cells. Although EV is less abundant than MV, it plays a vital role in dissemination within and between hosts [64].

## 3. Target Proteins for Specific mAb-Based Therapy Against Orthopoxviruses

The proteome of OPVs consists of approximately 200 proteins [65]. About half of these proteins are conserved across all OPV species and are essential for the formation of infectious MV and EV virions [66]. Disruption of any of these conserved proteins leads to a significant reduction in progeny production or complete loss of viral viability. The functions of these conserved proteins encoded in the central region of the genome are well-studied, making them optimal targets for specific therapies against OPVs. In the context of monoclonal antibody-based therapies, structural and membrane proteins are deemed the most suitable targets. However, it is important to note that the immune response to OPV infection also generates antibodies against secreted viral proteins [67,68].

Another important component of the OPV proteome includes proteins derived from non-conserved segments of the genome, which influence virulence and host range [69]. These proteins encompass soluble receptors involved in critical cell signaling pathways and those that modulate immune response activities. Additionally, non-secreted proteins containing domains such as ANK/PRANC, BTB/Kelth, Bcl, SPI, and others play a role in determining viral replication levels in various tissues and transmission between hosts [70,71]. Studies have shown that the deletion of host genes can induce programmed cell death or inhibit virus morphogenesis in specific cell cultures [72,73]. Moreover, this deletion can prevent acute infections of MPXV and VACV in susceptible animal models [74,75], highlighting these proteins as potential targets for anti-smallpox therapies.

However, targeting these proteins with antibody preparations presents challenges due to the high variability among OPV species and limited understanding of their functions [76]. Currently, there is no clear correlation between the number of genes encoding host range proteins and the diversity of animal hosts [77]. Some proteins can compensate for each other’s functions by directly interacting with the target protein or at various steps within signaling pathways, while certain orthologous proteins may exhibit narrow host specificity [78].

Our analysis of known neutralizing monoclonal antibodies against OPVs indicates that the primary targets for mAbs are conserved structural proteins B5 and A33 from the EV form, along with A27, D8, H3, and L1 from the MV form of VACV or their orthologs (Table 1). Nevertheless, other structural and non-structural conserved proteins may also be considered potential targets for broad-neutralizing antibodies against human-pathogenic OPVs.

## 4. Monoclonal Antibodies

To combat OPV infections, particularly those caused by MPXV, the CDC recommends the use of low molecular weight compounds and vaccinia immune globulin VIG derived from the pooled blood of vaccinated individuals [79]. These treatments were developed as emergency measures for severe infection. VIG has proven effective in mitigating side effects in adult vaccine recipients and, to a limited extent, in treating severe disease in children and immunocompromised patients [80]. However, the efficacy of VIG against other pathogenic OPVs remains unverified through direct studies, and its use is limited by inherent challenges associated with administering large protein quantities and standardization [81]. Additionally, the inability to rapidly procure sufficient doses limits the widespread application of VIG [68,82,83].

An alternative to VIG that avoids these drawbacks is the use of mAbs preparations, which have shown significant growth in the global pharmaceutical market over the past decade. Public health authorities have recognized mAbs as a potential treatment for infectious diseases, especially during the COVID-19 pandemic. Advances in mAb technology facilitate the enhancement of antibodies for specific applications, including the development of various recombinant antibody formats, antibody–drug conjugates, and the optimization of affinity, pharmacokinetics, and other pharmacological parameters [84].

Several mAbs (Table 1) demonstrated a high level of protection in model animals against lethal OPV infection. In most cases, an intramuscular dose of 45 to 100 µg of antibody was sufficient to achieve a protective effect, whereas VIG requires doses ranging from 1.25 mg to 5 mg—equivalent to 6000 IgG units/kg when administered to humans [85]. This suggests that therapies for OPV infections in humans may follow a similar pattern.

It is important to note that complete protection for the animals was not always achieved with mAbs, particularly in cases involving complicating factors such as immunodeficiency [86,87,88,89,90,91,92]. Employing combinations of different antibodies targeting one or more antigens can lead to enhanced protective outcomes. For instance, using a combination of mAbs VACV-301, VACV-249, MPXV-72, MPXV-26, VACV-22, and VACV-283 targeting MV and EV proteins provided complete protection for mice with combined immunodeficiency against lethal VACV infection, while individual antibodies resulted in 80–100% lethality [93]. However, combinations targeting only EV proteins appear to be less effective. For example, the combination of C6 and 8AH8AL antibodies did not achieve 100% protection in infected Balb/c mice, and weight loss was observed in animals regardless of whether the antibodies were administered together or separately [94].

Prophylactic administration of various combinations of mAbs 7D11, 1G10, and19C2 significantly increased protection in mice [95]. In SCID mice, mAbs h101 and hV26 targeting MV and EV, respectively, exhibited a dose-dependent protective response against lethal viral infection. The highest protection levels were observed when both antibodies were administered together [87,96]. Additionally, the B126 antibody effectively protected Balb/c mice from lethal viral infection and significantly accelerated weight regain when combined with the 11F7 antibody [91].

**Table 1:** Most effective mAbs against smallpox obtained over time. Key characteristics and potency against monkeypox.

MAb	Format	Isolation Technology	Epitope	Neutralizing Activity Analysis			First Ref.	Anti-MPXV Activity
				Activity	Method	Virus		
hMB621	IgG1	B-cell sorting (human)	B6 MPXV	IC50: 0.099 μg/mL (10% C′) Survival rate: 100% Weight loss: 10%	In vitro PRNT In vivo protection of BALB/c mice	VACV	Zhao 2024 [97]	Binding to epitope In vitro
hMB668	IgG1	“	“	IC50: 0.093 μg/mL (10% C′) Survival rate: 100% Weight loss: 10%	In vitro PRNT In vivo protection of BALB/c mice	“	“	“
VHH-1	VHH	phage display (alpaca VHH)	A35 MPXV	EC50: 0.010 ug/mL	ELISA	ND	Meng 2023 [98]	“
H8	VHH	Phage display (synth. library)	A29 MPXV	EC50: 1.5 nM	ELISA	“	Yu 2023 [99]	“
EV42	IgG1	Phage display (VH/VL primate B-cells)	A33	Plaque inhibition: 5 μg/mL Survival raten (day 2): 100% Weight loss: <20%	In vitro PRNT In vivo protection of BALB/c mice [100]	VACV_WR_-vFIRE ECTV Moscow	Noy-Porat 2023 [101]	Protecting In vivo Cast/EiJ mice [100]
MV33	IgG1	“	D8	IC50: 0.006 μg/mL Survival rate (day 2): 100% Weight loss: <20%	“	“	“	“
H2	IgG1	B-cells sorting (human)	A33	IC50: 3.6 μg/mL Survival rate: 80% Weight loss: 15%	In vitro PRNT In vivo protection of BALB/c mice	ECTV Moscow VACV_WR_	Gu 2022 [92]	Neutralizing In vitro
1.2.2.H9	IgG1	Phage display (VH/VL human B-cells)	D8	IC50: 0.013 µM (1% C′) Survival rate: 50% Weight loss: 0%	In vitro PRNT In vivo protection of NMRI mice	VACV Elstree	Diesterbeck 2021 [102]	ND
9C3	IgG2a	Hybridoma (BALB/c mice B-cells)	A14_71–90_ (conformat.)	IC80: 0.3 μg/mL (2% C′) IC40: 20 μg/mL Survival rate: 80% Weight loss: 5%	In vitro PRNT In vivo protection of SCID mice	ACAM2000	Meng 2018 [88]	ND
VACV-249	IgG1	Hybridoma (Human B-cells)	D8	IC50: 0.2 μg/mL (C′) Survival rate: 100% Weight loss: 15%	In vitro PRNT In vivo protection of C57BL/6 mice	VACV_WR_	Gilchuk 2016 [93]	Neutralizing In vitro
VACV-283	IgG1	“	B5	IC50: 0.7 μg/mL (C′) Survival rate: 100% Weight loss: 15%	“	“	“	“
VACV-22	IgG1	“	A33	IC50: 9.7 μg/mL (C′) Survival rate: 100% Weight loss: 10%	“	“	“	“
MPXV-72	IgG1	“	H3	IC50: 11.4 μg/mL (C′) Survival rate: 80% Weight loss: 20%	“	“	“	“
MPXV-26	IgG1	“	L1	IC50: 0.7 μg/mL (C′) Survival rate: 80% Weight loss: 20%	“	“	“	“
VACV-301	IgG3	“	A27	IC50: 0.1 μg/mL (C′) Survival rate: 100% Weight loss: 15%	*“*	“	“	“
1G6	IgG2a	Hybridoma (BALB/c mice B-cells)	A27_31–40_ (KREAIVKADE, (linear)	IC5: 20 μg/mL IC95: 20 μg/mL (C′) Survival rate: 80% Weight loss: 10%	In vitro PRNT In vivo protection of SCID mice	ACAM2000	Kaever 2016 [89]	“
A27D7	IgG2a	“	A33_149A–175A и 115B–177B_ (conformat.)	IC95: 10 μg/mL (10% C′) Survival rate: 100% Weight loss: 10%	In vitro PRNT In vivo protection of BALB/c mice	VACV_WR_	Matho 2015 [103]	Neutralizing In vitro
M12B9	IgG2a	“	L1_25–153_ (conformat.)	IC50: 0.032 μg/mL (1% C′) IC50: 0.4 μg/mL Survival rate: 50% Weight loss: >20%	In vitro PRNT In vivo protection of SCID mice	“	Kaever 2014 [90]	ND
11F7	IgG2a	“	A13_59–69_ (linear)	IC90: 0.03 μg/mL (2% C′) IC50: 0.2 μg/mL Survival rate: 80% Weight loss: 20%	In vitro PRNT In vivo protection of SCID mice	“	Xu 2011 [91]	Binding to epitope in silico
hV26	IgG1	KM mice B-cell hybridoma™	H3	IC50: 0.16 μg/mL (1% C′) IC50: 20 μg/mL (<20%). Survival rate: 0% Weight loss: 30%	In vitro PRNT [85] In vivo protection of SCID mice	“	McCausland 2010 [87]	ND
h104	IgG3	“	B5	IC50: 0.015 μg/mL (10% C′) IC50: 0.1 μg/mL Survival rate: 100% Weight loss: 0%	In vitro PRNT In vivo protection of BALB/c mice	“	Benhnia 2009 [104]	“
h101	IgG1	“	“	Survival rate: 40% Weight loss: 25%	In vivo protection of SCID mice [87]	“	“	“
B126	IgG2a	Hybridoma (BALB/c mice B-cells)	B5	IC50: 0.107 μg/mL (10% C′) IC50: 10 μg/mL Survival rate: 100% Weight loss: 5%	In vitro PRNT In vivo protection of BALB/cByJ mice	“	Benhnia 2009 [86]	“
				Survival rate: 0% Weight loss: 30%	In vivo protection of SCID mice	“	“	“
6C	IgG1	Phage display (VH/VL primate B-cells)	A33_99–185_ (conformat.)	IC75: 0.307 μg/mL (5% C′) IC50: ~121.5 μg/mL Survival rate: 100% Weight loss: 15%	In vitro PRNT [105] In vivo protection of BALB/c mice	“	Chen 2007 [94]	Binding to epitope in silico
8AH8AL	IgG1	“	B5_20–130_ (conformat.)	IC75: 0.142 μg/mL (5% C′) IC50: 121.5 μg/mL (25%) Survival rate: 100% Weight loss: 10%	In vitro PRNT [105] In vivo protection of BALB/c mice	“	Chen 2006 [106]	c8A variant protect NHP In vivo [107]
VMC-29	IgG1	Hybridoma (BALB/c mice B-cells)	B5_256–275_ (linear)	IC50: 25 μg/mL Survival rate: 100% Weight loss: 5%	In vitro PRNT In vivo protection of BALB/c mice [108]	“	Aldaz-Carroll 2005 [109]	Binding to epitope In vitro
10F10	ND	ND	A33 (conformat.)	IC50: 1/16384 A33 VACV IC50: 1/8192 A33o MPXV	ELISA	ND	Hooper 2000 [110]	“
1G10	IgG3	Hybridoma (BALB/c mice B-cells)	A33_D115/A118_ [111] (conformat.)	Survival rate: 100% Weight loss: 20%	In vitro PRNT In vivo protection of BALB/c mice [95]	VACV_WR_	Roper 1996 [112]	ND
7D11	IgG2a	ND	L1_37–158_ [113] (conformat.)	IC50: 0.0031 μg/mL Survival rate: 100% Weight loss: 20%	In vitro PRNT In vivo protection of BALB/c mice [95]	ND	Wolffe 1995 [114]	c7D11 protecting In vivo NHP [107]
19C2	IgG	Hybridoma (Rat B-cells)	B5	IC50: 3.1 ng/mL Survival rate: 100% Weight loss: 15%	In vitro PRNT In vivo protection of BALB/c mice [106]	VACV_WR_	Schmelz 1994 [115]	ND
5B4/ 2F2	IgG2a	Hybridoma (BALB/c mice B-cells)	A27_32–39_ (linear)	IC50: 12.5 μg/mL IC50: 1.6 μg/mL (1% C′) Survival rate: 100%	In vitro PRNT [116] In vivo protection of BALB/c mice	VACV MVA	Czerny 1990 [117]	Not binding to epitope In vitro
2G8/ 1E4	IgG3	“	A27_9–14_ (linear)	IC50: 200 μg/mL IC50: 12.5 μg/mL (1% C′)	In vitro PRNT	ECTV Munich 1	“	Binding to epitope In vitro
C3	IgG2a IgG2b	“	A27	IC50: 0.35 ng/mL Survival rate: 100% Weight loss: 5%	In vitro PRNT In vivo protection of BALB/c mice [118]	VACV_WR_	Rodriguez 1985 [119]	ND

“—repeated fragment of text; ND—no data; X% C′—PRNT in the presence of complement at a final concentration of X%; conformat.—conformational epitope.

Research has demonstrated that utilizing a combination of antibodies can significantly enhance protective efficacy, particularly when these antibodies target antigens from both forms of the virus.

A considerable proportion of engineered mAbs are directed towards membrane proteins such as MV L1, H3, D8, A13, A14, A27, and EV B5 and A33 proteins. These proteins are recognized as immunodominant antigens during vaccination or natural OPV infection [120,121]. Interestingly, EFC proteins are rarely targeted for specific mAb therapies despite their crucial involvement in viral cell entry. To date, only L1, A28, and H2 proteins have been identified as reliable targets of protective immunity during vaccination and passive antibody administration [122,123].

The presence of high neutralizing activity is anticipated in antibodies targeting A28 and L1 proteins even in the absence of complement [90,93]. Viral virulence factors and tropism proteins represent additional promising targets for therapeutic antibodies [124]. Immunization with the viral secreted type 1 interferon receptor (T1-IFNbp) has been shown to effectively prevent lethal VACV infections in mice [125], with similar protective effects observed following the administration of antibodies targeting T1-IFNbp [126]. Furthermore, antibodies directed against the complement control protein CPV have demonstrated comparable efficacy in reducing viral load and preventing the progression of acute infections [127].

The close antigenic relationship among human pathogenic OPVs suggests a significant potential for developing broadly neutralizing mAbs that can effectively neutralize related members within this group. Some of the antibodies listed in Table 1 have demonstrated high cross-neutralizing activity in vitro against OPVs, including MPXV-26, MPXV-72, and VACV-301 against CPXV and MPXV, as well as VACV-249 and VACV-283 against CPXV, and VACV-22 against MPXV [93]. Antibodies MPXV-26, MPXV-72, VACV-249, and VACV-283 exhibited a strong affinity for recombinant proteins or cell lysates infected with the VARV, suggesting their potential application in combating smallpox infection.

Additionally, mAbs 10F10 and 1G10 demonstrated specific binding to the VACV A33 antigen. Notably, only 10F10 interacted with a mutant VACV containing epitopes from CPXV and MPXV. It is interesting to note that the paratopes of both antibodies contain identical amino acids and demonstrate equivalent binding affinity towards VARV [128]. The structure of the 7D11-Fab:L1 complex has been thoroughly investigated [107], revealing that the antibody binds to a conformational epitope, with the amino acid residues of this epitope being shared among L1 proteins from CPXV, MPXV, and VARV. Furthermore, an analysis of the interaction interface between antibody M12B9 and L1 showed complete correspondence among the amino acid residues of the epitope for CPXV, MPXV, and VARV [90]. Antibody 11F7 displayed a high affinity for a linear epitope (59-ISSLYNLVKSS-69) within protein A13, which is conserved across all OPVs. This characteristic makes it a promising candidate for the therapy of OPV infections caused by CPXV, MPXV, and VARV [114]. Antibodies 6C and 8AH8AL demonstrated in vitro neutralizing activity against the VARV. The specificity of these antibodies to MPXV was confirmed through in silico analysis [94,106]. Prophylactic administration of a combination of antibodies c7D11 and c8A provided protection to non-human primates against MPXV infection [107]. The therapeutic potential of antibodies H2 [92] and VMC-29 [108] against infections caused by MPXV and VARV has been established based on their ability to bind to corresponding orthologous proteins. Antibody A27D7 bounds with high affinity to antigen A33 of VACV, which contains single alanine substitution and Gln117Lys/Leu118Ser substitutions corresponding to the epitopes of CPXV and MPXV [103]. A complete correlation between the amino acids of VACV and VARV, which play a role in the interaction between antigen and antibody, suggests that this antibody could be utilized as a countermeasure against smallpox infection.

For a panel consisting of six groups of antibodies [117,129] targeting linear epitopes of protein A27, regions 1A, 1B, and 4 were identified as targets for antibodies that neutralize VACV in a complement-dependent manner. Among the various antibodies analyzed, only 5B4/2F2 and 1B3/A11 selectively bound to regions 1A and 2, provided protection against lethal VACV infection in mice. However, these antibodies were unable to interact with MPXV, leaving their therapeutic potential for the VARV uncertain. Of particular interest is the neutralizing antibody 2G8/1E4 targeting the highly conserved region 4 (9-DDDLAI-14) that is identical in 372 of 391 members of the OPV genus. Although this antibody has not been tested in vivo, preliminary assays indicated its high binding affinity to the corresponding antigens [116].

The methods employed to obtain the listed mAbs (Table 1) differ significantly in terms of their sources and isolation techniques. Some mAbs were derived from immune repertoires following vaccination or incidental infections, while others were generated through immunization with live viruses or recombinant proteins. Notably, our analysis revealed no correlation between the efficacy of an antibody and its production method. Given that sera collected after multiple immunizations or severe viral infections demonstrate enhanced neutralizing activity, it is reasonable to anticipate that antibodies isolated from such donors would exhibit a high level of protective efficacy [130,131].

The potential of subunit vaccines in generating antibodies warrants particular attention. This approach not only enables precise customization of antigenic structures but also induces neutralizing antibody responses that, under certain conditions, are comparable to those elicited by live vaccines. Furthermore, subunit vaccines offer advantages such as eliminating the need for specific animal housing and allowing for rapid adaptation to emerging viral strains, as demonstrated in recent advancements in vaccine developments [123,132,133,134,135].

The majority of the antibodies obtained exhibit dose-dependent viral neutralizing activity in vitro, which is significantly enhanced in the presence of complement [86,87,88,89,90,91,93,94,102,103,104]. Neutralizing activity in vitro and in vivo depends largely on the number of antigen-recognition modules in the antibody molecule. For instance, different formats of the anti-L1 mAb 7D11 exhibited a remarkable 100-fold increase in neutralizing activity when comparing the F(ab’)2 formats to the Fab format alone [114]. Similarly, the full-length variant of mAb H2, which targets A33, demonstrated greater functional activity in vitro compared to its scFv counterparts [92]. In a comparison of various formats of the D8-targeting mAb 1.2.2.H9, including scFv-Fc, scFv and full-length IgG, it was found that neutralizing activity in vitro depended on the Fc region. However, there was no observed correlation between the different antibody formats and the level of protection they provided to immunocompetent mice against lethal viral infections [102]. It is evident that the effector functions of antibodies are an essential part of the humoral immune response against OPV infections. These functions rely on the activation of the complement system and interactions with receptors of innate and adaptive immune systems [136]. The significant contribution of the complement system to the induction of protective immunity against OPVs has been confirmed through experiments involving complement depletion, both in vitro and in vivo [86,137] as well as through various antibody formats. Notably, it has been shown that the ability of antibodies to engage with the complement system is influenced by their isotype. This was evidenced by a marked increase in neutralizing activity when using the constant regions of human IgG1 or IgG3, as well as mouse IgG2a, IgG2b, and IgG3 [86,89,104].

This underscores the importance of rational design approaches for antibody formats or Fc fragment engineering, which may enhance the effectiveness of therapeutic antibodies in OPV treatment in the near future.

## 5. Animal Models of Human OPV Infections

To evaluate the effectiveness of therapeutic agents and antibodies, it is essential to select a laboratory animal model that accurately reflects the process of infection development. Choosing an appropriate model is crucial for understanding the pathogenesis of the disease and for testing potential treatments. By utilizing models that closely mimic human responses to orthopoxvirus infections, researchers can gain valuable insights into the efficacy of therapeutic interventions, ultimately leading to better outcomes in clinical settings.

Animal models, varying in size from large to small, have been developed to study the pathogenesis of human OPV infections, including VARV, MPXV, CPXV, and VACV. These models exhibit characteristics that reflect certain aspects of smallpox progression in humans [138,139]. While VACV and ectromelia virus are considered surrogate models for MPXV and VARV and cause similar clinical features, they have a number of important differences. Therefore, it is difficult to regard them as fully adequate models. Specifically, both MPXV and VARV due to their similar clinical features also exhibit several important differences, making it challenging to regard them as fully adequate models. Specifically, both MPXV and VARV effectively utilize innate immune cells for their spread [140,141], with only MPXV causing significant lymphadenopathy [142]. In contrast, the ectromelia virus naturally enters the body through the skin of the paw. Although this entry route may not be critical for the experimental design, it is essential for interpreting results, as it influences the mechanism of spread within the organism, particularly concerning vector cells, which is not considered in this surrogate model.

The infection processes initiated by MPXV and especially VARV in non-human primates and small animals have been thoroughly investigated. Key characteristics such as tissue morphology changes, viral particle entry and spread within the body, and viral load in specific organs, have been well documented [143,144,145,146]. However, it should be noted that these data do not provide a comprehensive understanding of the pathophysiology of human infection caused by MPXV. The lack of information on viral load and degenerative changes in internal organs of MPXV-infected individuals, as well as the limited knowledge about VARV due to the absence of modern diagnostic methods, hampers a thorough analysis [144].

The initiation of persistent infection with MPXV and, in particular, with VARV, VARV often requires administering high doses of the virus, sometimes intravenously. However, this approach does not accurately reflect the natural route of infection and primary viremia [144,147,148,149]. In some animal models, viral infection is attainable only under immunodeficient conditions. For instance, infection of wild-type mice (Mus musculus) with VARV does not result in weight loss or other clinical manifestations even at doses up to 10^6^ particles. Conversely, CAST/EiJ mice, which carry a recessive phenotype of the interferon-gamma receptor, can succumb to infection even at a low dosage of 100 particles. Notably, CAST/EiJ mice exhibited a significant difference in the virulence between mutant and wild-type MPXV [150], but they did not show rashes regardless of the administration method or virus dose [151]. Another limitation in studying MPXV pathogenesis in mice is the development of systemic infection due to an inadequate innate immune response [152], particularly characterized by low levels of INF-gamma release upon infection [146]. Additionally, certain animal models—especially CAST and BALB/c mice—exhibit heightened susceptibility to VACV and some non-human primate species to CPXV compared to MPXV [153,154].

Among available models, black-tailed prairie dogs (*Cynomys ludovicianus*) are considered most suitable for studying MPXV due to their viral infection development and spread patterns that closely resemble those observed in non-human primates [155,156]. However, these species do not breed well in captivity, resulting in highly heterogeneous populations. African dormice demonstrate the highest susceptibility to MPXV, exhibiting indications of infection following exposure to low doses of the virus. Nevertheless, notable discrepancies exist between viral dissemination and accumulation patterns observed in this model when compared to other animal species [146].

In summary, these observations highlight the need for a critical evaluation of results obtained from animal models. Furthermore, integrating findings from multiple animals is essential for achieving a comprehensive understanding of the infectious process observed in humans. To study the complex molecular interactions and assess the contribution of viral proteins to the development of infection, it is necessary to utilize an animal model that closely resembles human molecular organization [157].

The most reliable study model for all OPVs is humanized animals, such as immunocompetent small Hu-CD34+ and Hu-CD34+/BLT humanized mouse models. These models replicate the smallpox infection pattern seen in humans and have shown good concordance in studies of progressive VARV infection [158].

Using single animal models, including small animals [159,160] and non-human primates (NHPs) [138,161] is more suitable for studying antibody drugs and low molecular weight antiviral agents targeting conserved proteins [162,163]. Evaluating changes in the viral load across organisms and an organism’s response to drug administration is sufficient to determine the effectiveness of therapy. Nevertheless, it is important to consider the potential species-specific differences in the kinetics of distribution and metabolism of the drug under investigation [156]. Of course, in cases where suitable animal models are unavailable, human cell models can be used to study antiviral drugs, such as mAbs, that influence the infection process of any potentially dangerous OPVs for humans.

## 6. Conclusions

The monkeypox virus has been rapidly spreading worldwide since 2022, necessitating increased attention from researchers and heightened readiness within healthcare systems. An effective response strategy to this viral threat involves assessing the applicability of existing diagnostic, therapeutic, and preventive measures, as well as identifying areas for improvement. To address this need, our study analyzes research focused on the development of virus-neutralizing monoclonal antibodies against human-pathogenic orthopoxviruses, particularly those with therapeutic potential.

A primary research objective remains the development of broadly neutralizing antibodies against human-pathogenic orthopoxviruses. This approach is preferable for covering a wider spectrum of viruses and is feasible due to the high conservation of immunodominant viral proteins within the orthopoxvirus family. While existing monoclonal antibodies primarily target the conserved structural proteins B5 and A33 of the extracellular enveloped virus (EV) form, and the A27, D8, H3, and L1 proteins of the mature virion (MV) form of vaccinia virus (VACV) or their orthologs, investigations into other structural proteins and secreted immunomodulators as potential targets are ongoing. The development of effective therapeutic agents is further complicated by the necessity of including antibodies that target both EV and MV forms of the virus to ensure comprehensive protection.

Many previously developed monoclonal antibodies have already been tested against MPXV in vitro and in vivo, yielding promising results. However, further progress in creating monoclonal antibody-based therapies is hindered by several limitations. Key challenges include the availability of suitable models that meet researchers’ requirements, as well as ethical considerations and safety issues related to using live viruses. Utilizing non-human primates (NHP) and various small animal models can help simulate natural infection processes but still requires careful selection for each orthopoxvirus species. Additionally, studying the protective properties of antibodies in human populations presents a complex ethical dilemma directly related to health and safety.

Over the past few decades, more than thirty original monoclonal antibodies have been isolated against human-pathogenic orthopoxviruses. It can be reasonably presumed that they may serve as the basis for drugs with the potential to treat a variety of infections caused by VACV-like viruses, CPXV, MPXV, and VARV. Further development of these antibodies could lead to new therapeutic strategies that will be effective against a wide range of orthopoxviruses posing a threat to human health.

It is important to note that further research and clinical trials are necessary to confirm the efficacy and safety of these antibodies in real-world conditions. Collaboration between scientists, healthcare professionals, and regulatory bodies will be key to the successful implementation of these new therapeutic approaches. Ultimately, the development and deployment of effective antibodies against orthopoxviruses can significantly enhance our preparedness for potential outbreaks and provide a higher level of protection for the poulation.

## Data Availability

No new data were created or analyzed in this study.

## Abbreviations

The following abbreviations are used in this manuscript:

VARV	Variola virus
MPXV	Monkeypox virus
CPXV	Cowpox virus
VACV	Vaccinia virus
VACV-like	Vaccinia virus-like virus
mAb	Monoclonal antibodies
DNA	Deoxyribonucleic acid
RNA	Ribonucleic acid
dsDNA	Double strand deoxyribonucleic acid
AT-rich	Adenine/thymine rich nucleotide sequence
MV	Matured virion
EV	Envelope virion
EFC	Entry fusion complex
ANK/PRANC	Ankyrin-repeat and closely related to the F-box domain proteins
BTB/Kelch	Proteins that consist of Kelch-repeat, tramtrack, and bric-a-brac domains
Bcl	B-cell lymphoma family protein
SPI	Serpin inhibitor-like proteins
IgG	Immunoglobulin G class
IgA	Immunoglobulin A class
VHH	Nanobodies
VH/VL	Variable domains of immunoglobulin
Fab	Fragment antigen-binding region
scFv	Single-chain variable fragment
scFv-Fc	Two single-chain variable fragments fused to the constant fragment igg
PRNT	Plaque reduction neutralization test
ELISA	Enzyme-linked immunosorbent assay
IC50	Half maximal inhibition concentration
EC50	Half maximal effective concentration
